# Replication of Novel Zoonotic-Like Influenza A(H3N8) Virus in Ex Vivo Human Bronchus and Lung

**DOI:** 10.3201/eid2906.221680

**Published:** 2023-06

**Authors:** Kenrie P.Y. Hui, John C.W. Ho, Ka-Chun Ng, Samuel M.S. Cheng, Ko-Yung Sit, Timmy W.K. Au, Leo L.M. Poon, John M. Nicholls, Malik Peiris, Michael C.W. Chan

**Affiliations:** The University of Hong Kong School of Public Health, Hong Kong, China (K.P.Y. Hui, J.C.W. Ho, K.-C. Ng, S.M.S. Cheng, L.L.M. Poon, M. Peiris, M.C.W. Chan);; Centre for Immunology and Infection, Hong Kong (K.P.Y. Hui, J.C.W. Ho, L.L.M. Poon, M. Peiris, M.C.W. Chan);; The University of Hong Kong Division of Cardiothoracic Surgery, Hong Kong (K.-Y. Sit, T.W.K. Au);; The University of Hong Kong School of Clinical Medicine, Hong Kong (J.M. Nicholls)

**Keywords:** influenza, H3N8, risk assessment, human bronchus, human lung, influenza A virus, avian influenza, zoonoses, China

## Abstract

Human infection with avian influenza A(H3N8) virus is uncommon but can lead to acute respiratory distress syndrome. In explant cultures of the human bronchus and lung, novel H3N8 virus showed limited replication efficiency in bronchial and lung tissue but had a higher replication than avian H3N8 virus in lung tissue.

Avian influenza viruses (AIVs) with reassortments between AIVs from domestic poultry and wild birds sporadically cross species barriers, leading to human infections. Viruses with internal genes of H9N2, hemagglutinin, and neuraminidase acquired from wild birds constitute the zoonotic H5N1, H7N9, and H10N8 viruses (*1*–*3*) and can lead to severe influenza.

In 2022, two human infections with novel influenza A(H3N8) viruses were reported in Henan and Hunan Province, China (*4*,*5*). The first case was identified in a 4-year-old boy with acute respiratory distress syndrome, and the second case occurred in a 5-year-old boy with mild disease. Phylogenetic analysis revealed that the novel H3N8 viruses were triple reassortments containing the Eurasian avian H3 gene of wild-bird origin, the North American avian N8 gene derived from the wild bird AIV, and G57 genotype H9N2 internal genes from AIVs found in poultry in China (*6*,*7*). H3N8 viruses that are genetically similar to the zoonotic H3N8 viruses reported in China (*4*,*5*) have been isolated in poultry markets in Hong Kong, China (*8*). Those novel avian H3N8 viruses are antigenically distant from contemporary human influenza A(H3N2) viruses, and little cross-reactive immunity to these chicken H3N8 viruses exists in the human population (*8*). We assessed the replication of the novel influenza A(H3N8) virus in human ex vivo bronchus and lung tissues (Appendix).

## The Study

**Figure 1:**
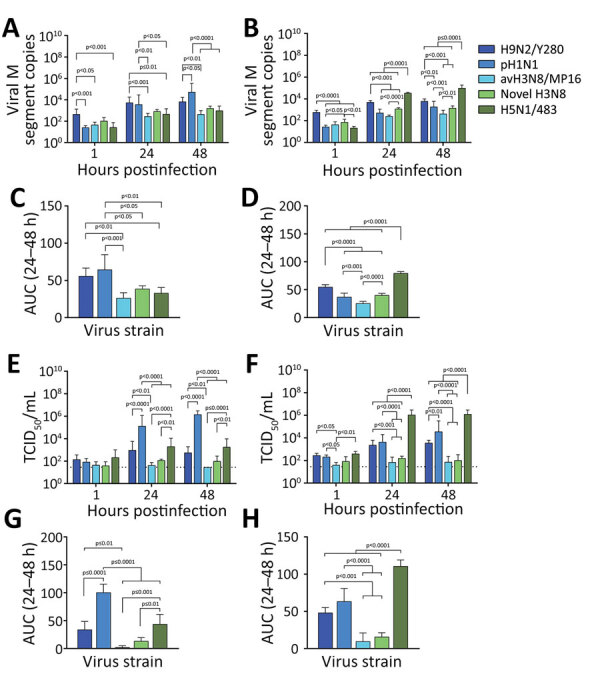
Comparative replication competence of zoonotic-like influenza A(H3N8) viruses isolated from chicken and other human and avian viruses in ex vivo cultures of human bronchus and lung tissue. Viral M segment RNA copies (A, B) and viral titers (E, F) in culture supernatants were collected at 1, 24, and 48 hours postinfection with H9N2/Y280, pH1N1, avH3N8/MP16, novel H3N8, or H5N1/483 viruses and measured by quantitative reverse transcription PCR (A, B) and TCID_50_ (E, F). C, D) Viral load from panels A and B by virus strain. G, H) Viral titers from panels E and F by virus strain. Data are geometric mean +SD. Statistical analysis was performed using 2-way (A, B, E, F)or 1-way (C, D, G, H) analysis of variance followed by Tukey posttest; p<0.05 was considered to be statistically significant. Detailed information on viruses used in study is provided in the Appendix. AUC, area under the curve; M, matrix; TCID_50_, 50% tissue culture infectious dose.

The viruses used in this study were H9N2/Y280, pH1N1, avH3N8/MP16, novel H3N8, and H5N1/483 (Appendix Table 1). The novel H3N8 virus was isolated from chickens and is genetically closely related to the virus causing zoonotic human disease in China (A/Henan/4-10CNIC/2022/H3N8) (*8*). Their hemagglutinin genes share a 99.1% similarity, and the neuraminidase genes share a 98.7% similarity. The avH3N8 virus was isolated from wild bird droppings in Mai Po, Hong Kong, and is genetically unrelated to the virus causing zoonotic disease in China. The novel H3N8 virus failed to propagate in Madin-Darby canine kidney (MDCK) cells but could be propagated in eggs and titrated in chicken embryo fibroblasts (DF-1), whereas the other strains could be propagated and titrated in MDCK cells. We performed titration in cells that support the replication of the influenza A viruses rather than in all DF-1 cells, because pH1N1 virus did not replicate in DF-1 cells. We investigated the virus replication kinetics by measuring viral matrix protein segment RNA in culture supernatants using real-time quantitative reverse transcription PCR and 50% tissue culture infectious dose (TCID_50_) assay for infectious virus titers (Figure 1).

In bronchial tissues, pH1N1 virus had a higher level of viral RNA than did avH3N8, novel H3N8, and H5N1, whereas levels of viral RNA of H9N2 virus were higher than those of avH3N8 and H5N1 virus (Figure 1, panels A, C). The viral RNA levels of avH3N8, novel H3N8, and H5N1 virus were similar. The viral RNA level of H5N1 virus was the highest among all the tested strains in human lung tissues, followed by H9N2 (Figure 1, panels B, D). Viral RNA levels of novel H3N8 and pH1N1 viruses were higher than those of avH3N8 virus. Measurement of viral RNA using quantitative reverse transcription PCR is sensitive, but it cannot distinguish defective viral particles from infectious ones. Therefore, we performed the TCID_50_ assay to monitor the infectious viral titers.

As expected, replication of pH1N1 virus was the highest among all tested strains in human bronchial tissues, in both viral titers and area under the curve values (Figure 1, panels E, G). The titers of H5N1 virus were similar to those of H9N2 virus, whereas H5N1 virus had higher replication competence than did avH3N8 and novel H3N8 viruses. We observed a discrepant trend between viral RNA copies and infectious titers for H9N2. Viral RNA levels of H9N2 were similar to those of pH1N1 (Figure 1, panels A, C), but the infectious titers of H9N2 virus were significantly lower than that of pH1N1 in bronchus (Figure 1, panels E, G).

In lung tissue, H5N1 virus had the highest replication of all strains tested (Figure 1, panels F, H). Similar to pH1N1 virus, H9N2 had higher titers than the 2 H3N8 viruses in lung tissues. avH3N8 had the lowest titer measured by TCID_50_. The novel H3N8 virus replicated poorly in mammalian MDCK cells but replicated efficiently in DF-1 avian cells (Appendix Figure). Those findings imply that the novel H3N8 virus has not yet adapted to mammal hosts, which was confirmed by limited replication in human bronchial and lung tissues (Figure 1, panels E, F).

**Figure 2:**
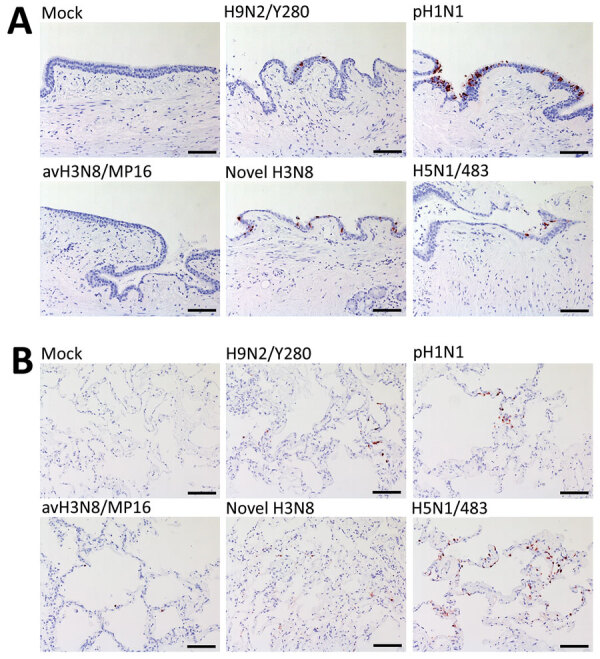
Tissue tropism of influenza A viruses in ex vivo cultures of human bronchus and lung tissue. Immunohistochemical staining of influenza A nucleoprotein in ex vivo cultures of human bronchial tissues (A) and lung tissues (B) at 48 hours postinfection with H9N2/Y280, pH1N1, H3N8/MP16, novel H3N8, and H5N1/483 viruses. Positive cells are indicated by red-brown color. Images are representative of 3 individual donors. Scale bar indicates 100 μm. Detailed information on viruses used in study is provided in the Appendix .

We fixed infected tissues at 48 hours postinfection and stained them for influenza A nucleoprotein immunohistochemistry (Appendix). Consistent with TCID_50_ findings, bronchial tissues infected with pH1N1 had the most extensive distribution of viral antigen, whereas we observed moderate levels of viral antigen staining in tissues infected with novel H3N8, H9N2, and H5N1 virus. No viral antigen staining was observed in tissues infected with avH3N8 (Figure 2, panel A). In the lung, H5N1 virus infection demonstrated the most extensive viral antigen staining, followed by infection with pH1N1, novel H3N8, and avH3N8 virus, which demonstrated the least extensive staining (Figure 2, panel B).

The discrepancy between the viral load in RNA copies and TCID_50_ titers of H9N2 and avH3N8 infection suggests that infection with those viruses might produce high levels of defective particles that cannot be detected by TCID_50_ assay. Immunohistochemistry staining of viral antigen serves as alternative evidence of virus replication in human tissues. The staining correlates more with TCID_50_ results than with viral RNA analysis for all the viruses.

Amino acid comparisons of the novel H3N8 and avH3N8 viruses demonstrated that they shared the same stalk length in the NA gene but did not have the G228S mutation that enhances binding to mammalian receptors (Appendix Table 2). The internal genes of the novel H3N8 virus were reassorted from H9N2 virus, whereas the internal genes of the avH3N8 came from H3N8, H6N1, H6N2, H3N8, H1N1, and H7N1 (Table). Neither virus had the E627K mutation in polymerase basic 2 that confers mammal adaptation, virulence, and transmissibility. The novel H3N8 virus had the A588V mutation in polymerase basic 2 that promotes mammal adaptation, but avian H3N8 virus did not have this mutation. This difference might contribute to higher replication of the novel H3N8 virus in human lung tissue. The S31N mutation found in the matrix protein 2 of the novel H3N8 virus provided adamantane resistance.

**Table:** Source of gene segments of novel and avian influenza A(H3N8) viruses*

**Gene segment**	Novel H3N8	avH3N8/MP16
**Polymerase basic 2**	H9N2	H3N8
**Polymerase basic 1**	H9N2	H6N1
**Polymerase acidic**	H9N2	H6N2
**Hemagglutinin**	H3N8	H3N8
**Nucleoprotein**	H9N2	H3N8
**Neuraminidase**	H3N8	H3N8
**Matrix**	H9N2	H1N1
**Nonstructural**	H9N2	H7N1

*Detailed information on viruses used in study is provided in the Appendix.

## Conclusions

Although zoonotic H3N8 viruses have a dual receptor-binding affinity of α-2,3 and α-2,6 receptors (*7*), our findings show that this factor does not confer an advantage for replication in human bronchial tissue. Our findings demonstrated inefficient replication of the novel H3N8 virus in human bronchial tissues, which implies limited efficiency to transmit among humans. This finding is in line with a recent serologic surveillance study in which no poultry workers were positive for antibodies for the novel H3N8 virus (*7*), and only 2 human cases have been documented since April 2022 (*4*,*5*). The moderate replication ability of the novel H3N8 virus in human lung tissue suggests that the virus causes less severe disease than H5N1 virus.

In summary, our findings suggest that the zoonotic-like avian H3N8 virus has limited efficiency for human-to-human transmission and, at present, is unlikely to cause severe disease in humans. However, the limited cross-reactive immunity against this novel H3N8 virus in the human population (*8*) and the emergence of novel H3N8 viruses by continuous reassortment between AIVs in wild birds and poultry demonstrate that the zoonotic and pandemic potential of avian H3N8 viruses should be closely monitored.

Appendix Additional information about replication of novel zoonotic-like influenza A(H3N8) in ex vivo human bronchus and lung
