# Supplementary material for: Replication of Novel Zoonotic-Like Influenza A(H3N8) Virus in Ex Vivo Human Bronchus and Lung
Source: Emerg Infect Dis. 2023 Jun;29(6):1210–4. doi: 10.3201/eid2906.221680 (PMC10202868; doi:10.3201/eid2906.221680)
Supplement: Appendix — Additional information about replication of novel zoonotic-like influenza A(H3N8) in ex vivo human bronchus and lung [file 22-1680-Techapp-s1.pdf]

Article DOI: https://doi.org/10.3201/eid2906.221680

EID cannot ensure accessibility for supplementary materials supplied by authors. Readers who have difficulty accessing supplementary content should contact the authors for assistance.

Replication of Novel Zoonotic-Like Influenza A(H3N8) Virus in Ex Vivo Human Bronchus and Lung

Appendix

Materials and Methods

Viruses

In addition to the zoonotic-like influenza H3N8 (A/Env/Hong_Kong/MKT_AB_13cp/2022, novel H3N8) which is a chicken virus, an avian virus isolated from wild bird droppings in Mai Po, Hong Kong (A/Env/Hong Kong/MP16_265/2016, avH3N8/MP16), a highly pathogenic avian influenza (HPAI) H5N1 virus (A/Hong Kong/483/1997, H5N1/483) isolated from a fatal human infection, a 2009 pandemic influenza virus (A/Hong Kong/415742/2009, pH1N1) isolated from a patient in Hong Kong and a duck H9N2 virus (A/Duck/Hong Kong/Y280/97, H9N2/Y280) were used as controls. The novel H3N8 virus was propagated in eggs while all the other influenza viruses were passaged in Madin-Darby canine kidney (MDCK) cells. Virus stock was aliquoted and titrated to determine the plaque forming unit per mL (pfu/mL) in chicken embryo fibroblast (DF-1) cells for the novel H3N8 virus and MDCK cells for the other influenza viruses. The experiments were carried out in a Bio-safety level 3 (BSL-3) facility at the School of Public Health, LKS Faculty of Medicine, The University of Hong Kong.

Ex vivo cultures and infection of human bronchus and lung

Fresh non-tumor bronchus and lung tissues were obtained from patients undergoing elective surgery in Department of Surgery of Queen Mary Hospital and were removed as part of clinical care but surplus for routine diagnostic requirements as detailed previously (*1*). Sampling

of tissues is defined by convenience. Fragments of human tissues were infected with each virus at $1x10^6$ pfu/mL for 1 h at 37°C. Mock-infected tissue served as negative controls. The explants were washed three times with PBS and placed in culture medium (F-12K nutrient mixture with L-glutamine, and antibiotics) with or without a sterile surgical pathology sponge to establish an air-liquid interface condition in 24-well culture plates in a 37°C incubator with 5% CO_2. Infectious viral titers in culture supernatants were assessed at 1, 24, and 48 hours post-infection (hpi) by $TCID_{50}$ assay. Bronchus and lung tissues were fixed at 48 hpi in 10% formalin and processed for immunohistochemistry staining. Ethics approval of the use of human tissues was granted by the institutional review board of the University of Hong Kong and the hospital authority (approval number UW 20–862).

Viral titration by $TCID_{50}$ assay

A confluent 96-well tissue culture plate of DF-1 or MDCK cells was prepared 1 day before the virus titration ($TCID_{50}$) assay. Cells were washed once with PBS and replenished serum-free MEM medium supplemented with 100 units/ml penicillin and 100 µg/ml streptomycin and 2 µg/ml of tosylsulfonyl phenylalanylchloromethyl ketone treated trypsin for DF-1 and MDCK cells. Serial dilutions of virus supernatant, from 0.5 log to 7 log, were performed before adding the virus dilutions onto the plates in quadruplicate. The plates were observed for cytopathic effect daily. The endpoint of viral dilution leading to CPE in 50% of inoculated wells was estimated using the Karber method (*2*). AUC was calculated from the viral titers from different time points indicated in the y-axis.

Quantitative reverse transcription PCR assay

The viral RNA in 20 uL culture supernatants was extracted using the QIAamp Viral RNA Mini kit (Qiagen). RNA was reverse-transcribed by using random 6-mer primers with PrimeScript RT reagent Kit (Takara). Viral RNA copy number of target genes was performed using an ABI ViiA 7 real-time PCR system (Applied Biosystems). All procedures were performed according to the manufacturers' instructions as previously described (*3*,*4*).

Immunohistochemical staining

The human tissues were fixed with 10% formalin overnight, embedded in paraffin blocks and stained for influenza viral protein as previously described (*4*). Briefly, the paraffin-embedded tissues were sectioned, deparaffinised, digested with pronase and blocked with an

avidin/biotin blocking kit (Vector Labs, Burlingame, CA, USA). The sections were then stained with influenza A virus nucleoprotein-specific mouse monoclonal antibody HB65 (EVL, Woerden, The Netherlands) and a biotinylated secondary antibody. The bound antibodies were visualized with a Strep-ABC complex and an AEC substrate kit.

Statistical analysis

Experiments with the human ex vivo cultures were performed independently with five and six different donors of bronchus and lung, respectively. Results shown in figures are geometric mean (+/−SD). Area-under-the-curve (AUC) was calculated by integrating infectious virus titers at 24–48 hpi in ex vivo bronchus, lung tissues. The differences in log10-transformed viral titers and quantitative viral RNA of M segment between viruses and over time were compared using two-way ANOVA followed by a Tukey's multiple-comparison test using GraphPad Prism version 9.0.0. Comparison of AUC between viruses was calculated using one-way ANOVA followed by a Tukey's multiple-comparison test. Differences were considered significant at a *P* value less than 0.05.

References

1. Hui KP, Chan LL, Kuok DI, Mok CK, Yang ZF, Li RF, et al. Tropism and innate host responses of influenza A/H5N6 virus: an analysis of *ex vivo* and *in vitro* cultures of the human respiratory tract. Eur Respir J. 2017;49:1601710. PubMed https://doi.org/10.1183/13993003.01710-2016

2. Kärber G. Contribution to the collective treatment of pharmacological experimental series [in German]. Arch. Exp. Pathol. Pharmacol. 1931;162:480–3. PubMed https://doi.org/10.1007/BF01863914

3. Hui KPY, Ho JCW, Cheung MC, Ng KC, Ching RHH, Lai KL, et al. SARS-CoV-2 Omicron variant replication in human bronchus and lung ex vivo. Nature. 2022;603:715–20. PubMed https://doi.org/10.1038/s41586-022-04479-6

4. Hui KP, Chan LL, Kuok DI, Mok CK, Yang ZF, Li RF, et al. Tropism and innate host responses of influenza A/H5N6 virus: an analysis of *ex vivo* and *in vitro* cultures of the human respiratory tract. Eur Respir J. 2017;49:1601710. PubMed https://doi.org/10.1183/13993003.01710-2016

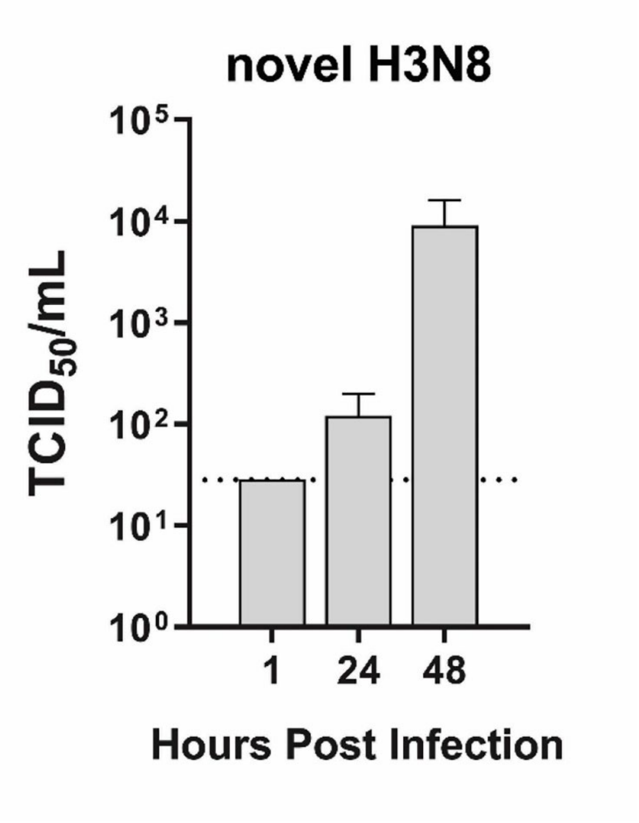

Appendix Figure. Novel H3N8 virus replication in DF-1 cells infected at MOI 0.01. $TCID_{50}$ assay was performed in DF-1 cells. Dotted line represents the detection limit of $TCID_{50}$ assay.

Appendix Table 1. Influenza A virus strains used

Virus	Strain name
H9N2/Y280	A/Duck/Hong Kong/Y280/97
pH1N1	A/Hong Kong/415742/2009
avH3N8/MP16	A/Env/Hong Kong/MP16_265/2016
Novel H3N8	A/Env/Hong_Kong/MKT_AB_13cp/2022
H5N1/483	A/Hong Kong/483/1997

Appendix Table 2. Amino acid comparison between the novel H3N8 (AB_13) and avian H3N8 (MP16)

HA1	amino acid position	2	6	8	19	45	57	58	70	83	92	102	112	119	124	128	131	137	145	159
A/Env/Hong_Kong/MKT_AB_13cp/2022		N	K	S	S	N	K	V	M	K	S	V	I	D	S	A	S	G	N	S
A/Env/Hong_Kong/MP16_265/2016_(H3N8)_ Anas_acuta		D	N	N	A	S	R	I	L	T	N	I	V	E	G	T	T	S	S	N
	amino acid position	160	163	172	214	228	257	260	264	275	312									
		S	L	Y	V	G	F	I	R	E	N									
		A	V	D	I	G	Y	M	K	D	T									
NA	amino acid position	14	20	22	26	30	37	38	39	43	44	49	50	59	61	62	69	71	74	75
A/Env/Hong_Kong/MKT_AB_13cp/2022		V	L	I	I	T	P	R	D	R	N	V	I	I	K	V	N	I	I	E
A/Env/Hong_Kong/MP16_265/2016_(H3N8)_ Anas_acuta		I	F	V	V	I	G	K	G	G	V	I	V	V	R	I	S	V	M	P
	amino acid position	76	77	78	79	80	81	82	89	92	125	154	161	191	198	209	235	237	253	254
		R	P	E	N	D	H	F	L	A	T	Y	I	I	A	I	E	F	A	F
		Y	W	K	E	G	T	Y	i	V	I	F	V	V	S	V	D	Y	I	Y
	amino acid position	257	260	263	265	266	267	283	311	329	355	375	376	377	381	383	388	389	392	397
		K	K	E	A	E	I	E	R	S	N	K	V	R	V	N	I	K	V	S
		S	R	G	R	D	V	D	K	A	T	R	I	K	T	T	V	R	I	L
	amino acid position	410	414	415	416	452	469													
		V	K	R	N	I	E													
		I	G	K	D	V	K													
PB2	amino acid position	588	627																	
A/Env/Hong_Kong/MKT_AB_13cp/2022		V	E																	
A/Env/Hong_Kong/MP16_265/2016_(H3N8)_ Anas_acuta		A	E																	
M2	amino acid position	31																		
A/Env/Hong_Kong/MKT_AB_13cp/2022		N																		
A/Env/Hong_Kong/MP16_265/2016_(H3N8)_ Anas_acuta		S																		
